# Daily Step Counts in Patients With Chronic Kidney Disease: A Systematic Review and Meta-Analysis of Observational Studies

**DOI:** 10.3389/fmed.2022.842423

**Published:** 2022-02-17

**Authors:** Fan Zhang, Yibo Ren, Hui Wang, Yan Bai, Liuyan Huang

**Affiliations:** ^1^Department of Nephrology, Longhua Hospital Shanghai University of Traditional Chinese Medicine, Shanghai, China; ^2^Department of Anorectal, Longhua Hospital Shanghai University of Traditional Chinese Medicine, Shanghai, China; ^3^Department of Cardiology, Longhua Hospital Shanghai University of Traditional Chinese Medicine, Shanghai, China

**Keywords:** chronic kidney disease, daily step counts, physical activity, systematic review, meta-analysis

## Abstract

**Background:**

Physical inactivity is an essential factor in the prognosis of patients with chronic kidney disease (CKD). Daily step count is a straightforward measure to assess physical activity levels. Understanding the step counts among different CKD stages is essential to change sedentary behavior.

**Objectives:**

This systematic review and meta-analysis aimed to investigate the daily step counts in patients with CKD at a different stage.

**Design:**

A systematic review and meta-analysis.

**Data Sources:**

The literature search was performed in PubMed, Embase, and Web of Science from inception to November 3rd, 2021.

**Review Methods:**

Observational studies (cross-sectional, case-control, or cohort studies) reported specific values of step counts in CKD patients by the wearable device were included. A random-effects model was used to pool the data. Subgroup analysis explored differences in outcomes by stage of CKD. Heterogeneity between studies was assessed using the χ^2^ test of Cochrane's *Q* statistic. A contour-enhanced funnel plot was conducted to investigate publication bias. Univariate and multivariate meta-regression was conducted to examine possible sources of heterogeneity.

**Results:**

Twenty-eight articles were identified and used for quantitative analysis. The result showed that the daily step count in patients with CKD was 4642.47 (95% *CI*: 4274.18–5010.76), and significantly lower than the healthy population. Subgroup analysis revealed that the step counts decreased before dialysis, dropped to a freezing point at the hemodialysis phase, and increased after kidney transplantation. Meta-regression analysis showed that daily step counts were relatively higher in the Americas or younger than 60 or kidney transplant recipients.

**Conclusion:**

The status of daily step counts in patients with CKD decreases with CKD severity and increases after kidney transplantation. Although studies have begun to focus on strategies to improve step counts in patients with CKD, future studies should focus more on step counts in pre-dialysis patients and changing their physically inactive lifestyle early to alleviate deteriorating renal function.

**Systematic Review Registration:**

https://www.crd.york.ac.uk/prospero/display_record.php?RecordID=291551, identifier: CRD42021291551.

## Introduction

Chronic kidney disease (CKD) has emerged as a major worldwide public health problem, with adverse physical, psychological, and economic outcomes (1). Being physically active is a healthy lifestyle considered a core part of the general and CKD populations (2). A recent Meta-analysis of a cohort study of patients with CKD showed that high physical activity was associated with a 14% reduction in all-cause mortality compared to low physical activity (3). Correspondingly, the UK Renal Association Clinical Practice Guideline recommends that patients with CKD engage in at least 30 min of moderate-intensity physical activity five times per week (4).

Walking is the most common physical activity for chronic diseases, including CKD (5). With the advent of pedometers, it has become feasible in the real world to accurately quantify the number of steps an individual walks each day. The number of daily steps reflects the amount of physical activity an individual is doing (6). Since the use of pedometers in medical research in the 1980s (7), numerous research studies have been conducted on the number of daily steps taken by individuals with CKD.

However, the mean or the median number of steps reported by different populations varies widely. Given the diversity of the results of these studies, this study evaluates the daily step counts in patients with CKD at a different stage by using a systematic review and meta-analysis to determine the differences between subgroups and provide a reference for promoting physical activity in patients with CKD.

## Methods

This systematic review has been reported according to the Preferred Reporting Items for Systematic Reviews and Meta-Analyses (PRISMA) criteria statement (8). The study protocol was registered with the International Prospective Register of Systematic Reviews (PROSPERO), and the registration number was CRD42021291551.

### Search Strategy

We systematically searched PubMed, Embase, and Web of Science from inception to November 3rd, 2021. Free-text words and medical subject heading (MeSH) terms were used. The whole search strategy is presented in Supplementary Material 1. Moreover, we manually retrieved a relevant reference of systematic reviews to search for additional potential studies.

### Eligibility Criteria

Published observation studies with the following criteria were considered eligible for inclusion in this meta-analysis: (1) participants: CKD patients. There is no limit to age and disease stage (pre-dialysis, peritoneal dialysis, hemodialysis, and kidney transplant recipients were eligible); (2) outcomes: the wearable device to measure the daily steps count; (3) studies: only observational study (cross-sectional, case-control, or cohort studies) designs were included; (4) studies reported specific values [mean (standard deviation, SD), median (quartile or range)] of patient steps were eligible. One literature was excluded if it met at least one of the following criteria: (1) patients affected by acute kidney failure; (2) clinical intervention trials, conference abstract, case reports, protocol, letters, commentaries, reviews, and editorials; (3) written in non-English.

### Literature Selection

Two authors (Yan Bai and Hui Wang) performed the literature selection independently, and a third author helped resolve any discrepancies if encountered. First, the titles/abstracts of all retrieved articles were reviewed, and studies that did not meet the inclusion criteria were excluded. Then, to determine if the articles meet final eligibility criteria, each author further reviewed the full text of the remaining studies.

### Data Extraction

Two independent authors (Yan Bai and Hui Wang) extracted the relevant information as follows: (1) first authors; (2) publication year; (3) sample size; (4) participant's age; (5) daily step counts; (6) assessment tool. We tried to contact the corresponding author for unclear data to determine whether to include this literature. Any disagreement about data extraction was resolved through consulting a third author.

For quantitative analysis, the mean, standard deviation (or standard error), and sample size were extracted from the included article. When results were presented in figures, data were extracted using the GetData software (getdata-graph-digitizer.com) (9–11). When results were described in terms of median, quartiles (or range) (12–20), data were calculated using the formula proposed by Luo et al. and Wan et al. (https://www.math.hkbu.edu.hk/~tongt/papers/median2mean.html) (21, 22). If data were reported for subgroups (e.g., male vs. female) (23–25), we calculated a pooled mean (standard deviation) by pooling the data of the two different subgroups (26). Furthermore, if a study reported results for different stages of CKD separately, both data were included in the meta-analysis.

### Risk of Bias

The quality of cross-sectional and longitudinal studies was assessed using the JBI Meta-Analysis of Statistics Assessment and Review Instrument (JBI-MAStARI) (27). The JBI-MAStARI includes eight questions. Two authors (Yan Bai and Hui Wang) performed the quality assessment procedure independently, with disagreements discussed by both authors or with the help of a third author.

### Data Synthesis and Statistical Analysis

Quantitative data synthesis was used to present the data extracted from each study. Given the potential heterogeneity across the included studies, a random-effects model was used with the DerSimonian-Laird method. Subgroup analysis explored differences in outcomes by stage of CKD.

Heterogeneity between studies was assessed using the χ^2^ test of Cochrane's Q statistic, and I-square estimates >75% were considered highly heterogeneous (28). Contour-enhanced funnel plots, the Egger's test, and the trim-and-fill method were conducted to investigate publication bias across studies.

Univariate and multivariate meta-regression were conducted to examine possible sources of heterogeneity using the following variables: region (Asia, Europe, and America), stage of CKD (pre-dialysis, peritoneal dialysis, hemodialysis, and kidney transplant recipients), samples (>100 and ≤100), bias (score < 7 and ≥7), age (<60 and ≥60), and measurement tools (Accelerometer, Armband, Pedometer, and Fitbit).

*P* < 0.05 was considered statistically significant. Stata software (version 12.0) was used to analyze the data.

## Results

### Search Results

**Figure 1:**
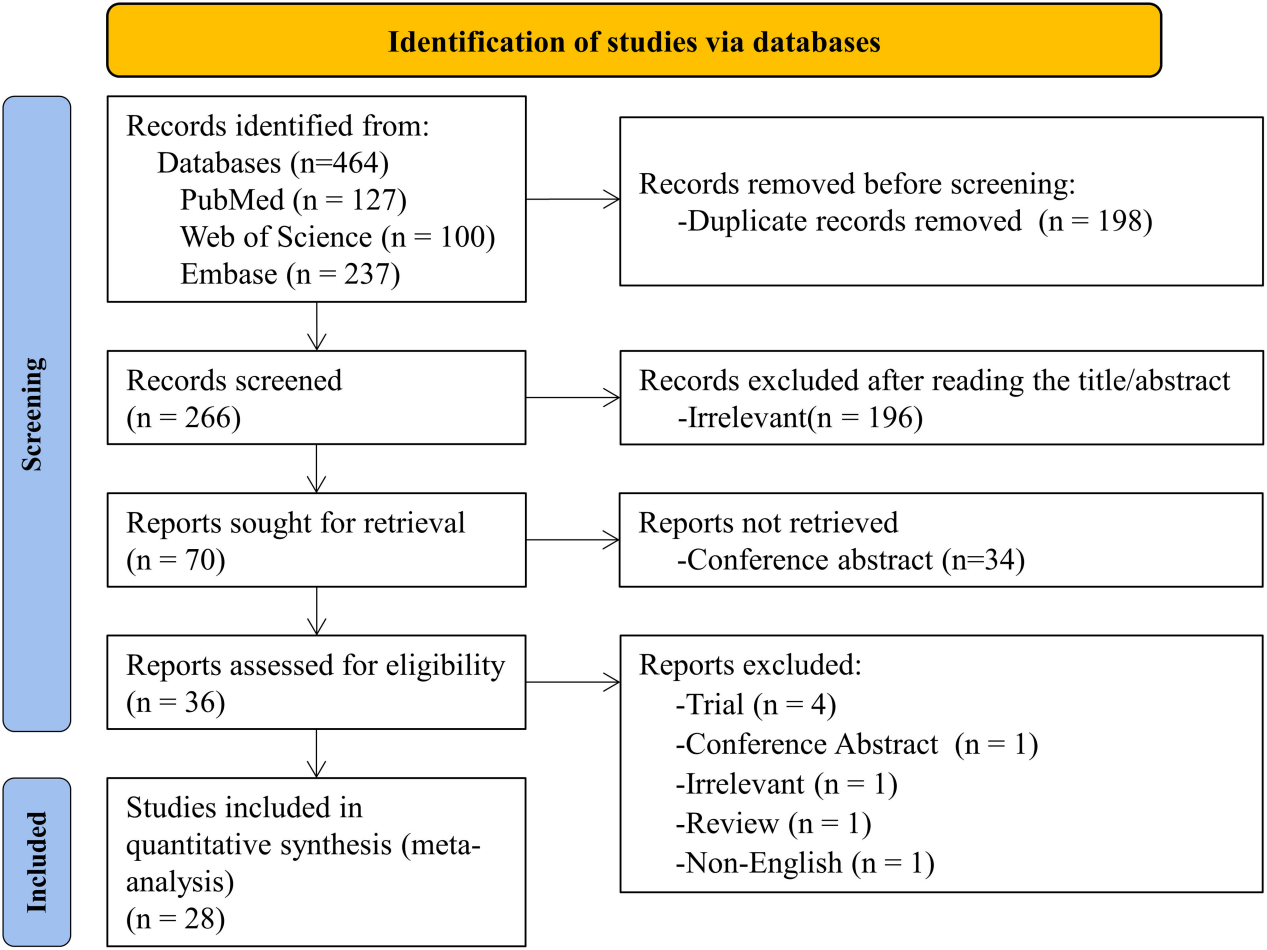
Flowchart showing the results of the selection process.

The initial search yielded 464 articles and abstracts, and 198 pieces were excluded due to duplication. After screening titles and abstracts, 196 irrelevant articles were excluded. The remaining 36 articles were searched in full text for review based on inclusion and exclusion criteria. Eight articles were excluded, and the reasons for exclusion were listed in Supplementary Material 2. Finally, 28 articles were identified and used for quantitative analysis (9–20, 23–25, 29–41). The flow chart is based on PRISMA 2020 Flow Diagram (8) (Figure 1).

### Study Characteristics and Participants

Hemodialysis patients were the most reported population. Study sample sizes ranged from 20 to 1,163 (Table 1), with a total sample size of 3,769. The age range across the studies was 15.1–70.9 years. Two studies used a longitudinal design, and 22 were cross-section studies, and four were cohort studies. The details of the quality assessment are shown in Supplementary Material 3.

**Table 1:** Characteristic of observation studies included in the meta-analysis.

**References**	**Design**	**Sample size**	**Participant's age (years)**	**Stage of CKD**	**Measurement**	**Daily step counts**
Cupisti et al. (29)	Cross-sectional study	50 (32 male; 18 female)	59 ± 13	HD	SenseWear™ Armband	HD: 5,584 ± 3,734 Normal subjects: 11,735 ± 5,130
Matsuzawa et al. (15)	Cross-sectional study	116 (58 male; 58 female)	68 (62, 74)	HD	Pedometer	3,208 (1,828, 4,481)
Cobo et al. (30)	Cross-sectional study	78 HD (51 male; 27 female) 64 PD (45 male; 19 female)	HD: 63 ± 12 PD: 62 ± 14	PD and HD	Pedometer	HD day: 2,274 ± 2,048 Non-HD day: 3,769 ± 3,370 PD: 4,839 ± 3,313
Brys et al. (18)	Cross-sectional study	37	63 ± 18	HD	SenseWear™ Armband	2,424 (892, 4,545)
Sheshadri et al. (13)	Cross-sectional study	48 (39 male; 9 female)	57 (52,65)	HD	Pedometer	2,631 (1,125, 5,278)
Cobo et al. (25)	Cross-sectional study	57	65 (49,80)	HD	Geonaute-onstep-400^®^ pedometer	Normal testosterone: 4,291 ± 3,225 Hypogonadism: 2,753 ± 1,784
Yamamoto et al. (31)	Retrospective cohort study	512 (299 male; 213 female)	65.4 ± 11.7	HD	Accelerometer	Total: 3,268 (1,749–5,195) HD day: 2,371 (1,203–4,240) Non-HD day: 3,752 (1,830–6,271)
Mafra et al. (23)	Cross-sectional study	24 (18 male; 6 female)	CRP <5: 67.0 ± 14.7 CRP>5: 69.0 ± 18.0	HD	SenseWear Pro2 Armband	CRP <5: 6,016 ± 3,752 CRP > 5: 2,801 ± 2,754 Normal subjects: 8,107 ± 5,419 CRP <5: HD day 4,748 ± 3,471 CRP <5: Non-HD day 7,000 ± 4,670 CRP > 5: HD day 1,810 ± 1,566 CRP > 5: non-HD day 2,148 ± 1,780
Dontje et al. (32)	Longitudinal study	28 (14 male; 14 female)	54.5 (IQR 15)	KTRs	SenseWear™ Armband	6,326 ± 2,906
D'Alessandro et al. (24)	Prospective cohort study	144 (120 male; 24 female)	Diabetic CKD: 71.5 ± 8.2 non-diabetic CKD: 71.6 ± 9	Stage 3b−4 CKD (eGFR: 29.8 ± 9.1 vs. 31.3 ± 10.9)	SenseWear™ Armband	Diabetic CKD: 3,580 ± 2,471 Non-diabetic CKD: 5,628 ± 4,143
Akber et al. (20)	Cross-sectional study	44 (22 male; 22 female)	15.1 ± 3.4	CKD stage 1–4 [eGFR: 40.5 (27.5, 77.0)]	Yamax Digi-walker SW-200 pedometer	6,218 (3,637, 9,829)
Panaye et al. (14)	Cross-sectional study	1163 (762 male; 401 female)	63 (51–75)	HD (n = 1100) PD (n = 63)	Pedometer	Total: 3,688 (1,866–6,271) HD: 3,693 (1,896–6,307) PD: 3,320 (1,478–5,926) HD day: 2,912 (1,439–5,232) Non-HD day: 4,054 (2,136–7,108)
Avesani et al. (19)	Cross-sectional study	134 (64 male; 70 female)	54.9 ± 15.9	HD	SenseWear Pro2 Armband	Total: 5,660 (73, 16,565) HD day: 4,620 (77, 13,957) Non-HD day: 5,544 (72, 18,220)
Hamiwka et al. (33)	Cross-sectional study	20 (8 male; 12 female)	14.3 ± 3.2	KTRs	Digi-walker SW200 pedometer	KTRs: 9,282 ± 4,666 Normal subjects: 11,449 ± 4,638
Williams et al. (9)	Cross-sectional study	29 (12 male; 17 female)	52 ± 14	HD	Fitbit^®^ Flex™ tracking bracelet	HD: 5,291 ± 2,338 HD day: 4351.14 ± 2266.12 (SE) Non-HD day: 6396.91 ± 4909.89 (SE)
Lou et al. (34)	Cross-sectional study	320 (120 male; 200 female)	58.60 ± 14.2	HD	OMRON pedometer	3725.92 ± 2663.47
Carvalho et al. (35)	Cross-sectional study	HD: 20 (11 male; 9 female) KTRs: 23 (11 male; 12 female)	HD: 47.3 ± 12.6 PD: 48.3 ± 10.3	HD KTRs	Triaxial accelerometer	KTRs: 9,705 ± 4,902 HD day: 6,962 ± 3,352 Non-HD day: 4,396 ± 2,034
Malhotra et al. (11)	Cross-sectional study	45 (26 male; 19 female)	61 ± 15	HD	Fitbit^®^ Charge 2 tracker	3,688 ± 2,730 HD day: 3165.47 ± 2455.63 (SE) Non-HD day: 4124.70 ± 3453.24 (SE)
Oishi et al. (36)	Cross-sectional study	38 (22 male; 16 female)	63.9 ± 10.8	PD	Multi-memory pedometer	4,367 ± 2,590
Han et al. (39)	Cross-sectional study	29 (16 male; 13 female)	53 ± 11	HD	Fitbit^®^ Flex	8,454 ± 4,087
Shibata et al. (37)	Cross-sectional study	24 (13 male; 11 female)	66.0 ± 8.2	HD	Lifestyle recording device	HD: 4,774 ± 2,845 Normal subjects: 8,696 ± 3,047
Katayama et al. (38)	Longitudinal study	71 (43 male; 28 female)	70.9 ± 10.6	HD	Tri-accelerometer	2445.7 ± 2018.3 HD day: 2370.6 ± 2044.6 Non-HD day: 2502.1 ± 2236.5
Han et al. (10)	Cross-sectional study	46 (23 male; 23 female)	54 ± 12.9	HD	Fitbit^®^ Flex	Total: 6,393 ± 3,550 HD day: 5642.42 ± 4369.68 (SE) Non-HD day: 6872.73 ± 4581.77 (SE)
Zhang et al. (12)	Cross-sectional study	174 (93 male; 81 female)	63.05 ± 12.29	HD	Digital pocket pedometer	Low muscle: 2,803 (824, 4,154) High muscle: 5,589 (4,659, 6,511)
Kittiskulnam et al. (17)	Cross-sectional study	60 (47 male; 13 female)	58.0 ± 12.7	HD	Pedometer	2630.5 (1270.7, 5137.0)
Raymond et al. (41)	Cross-sectional study	32 (14 male; 18 female)	Not reported	KTRs	Piezo SC-StepMX step pedometer	9,752 ± 3,685
Lunney et al. (16)	Prospective cohort study	46 (29 male; 17 female)	64 (47, 71)	HD	Fitbit tracker	3,133 (1,976, 5,097)
Matsuzawa et al. (40)	Prospective cohort study	282 (154 male; 128 female)	64.8 ± 10.6	HD	Accelerometer	3,920 ± 2,797 HD day: 3,099 ± 4,337 Non-HD day: 4,337 ± 3,160

*CKD, Chronic Kidney Disease; PD, Peritoneal Dialysis; HD, Hemodialysis; KTRs, Kidney Transplant Recipients; CRP, C-reaction Protein*.

### Overall Daily Step Counts Among Patients With CKD

**Figure 2:**
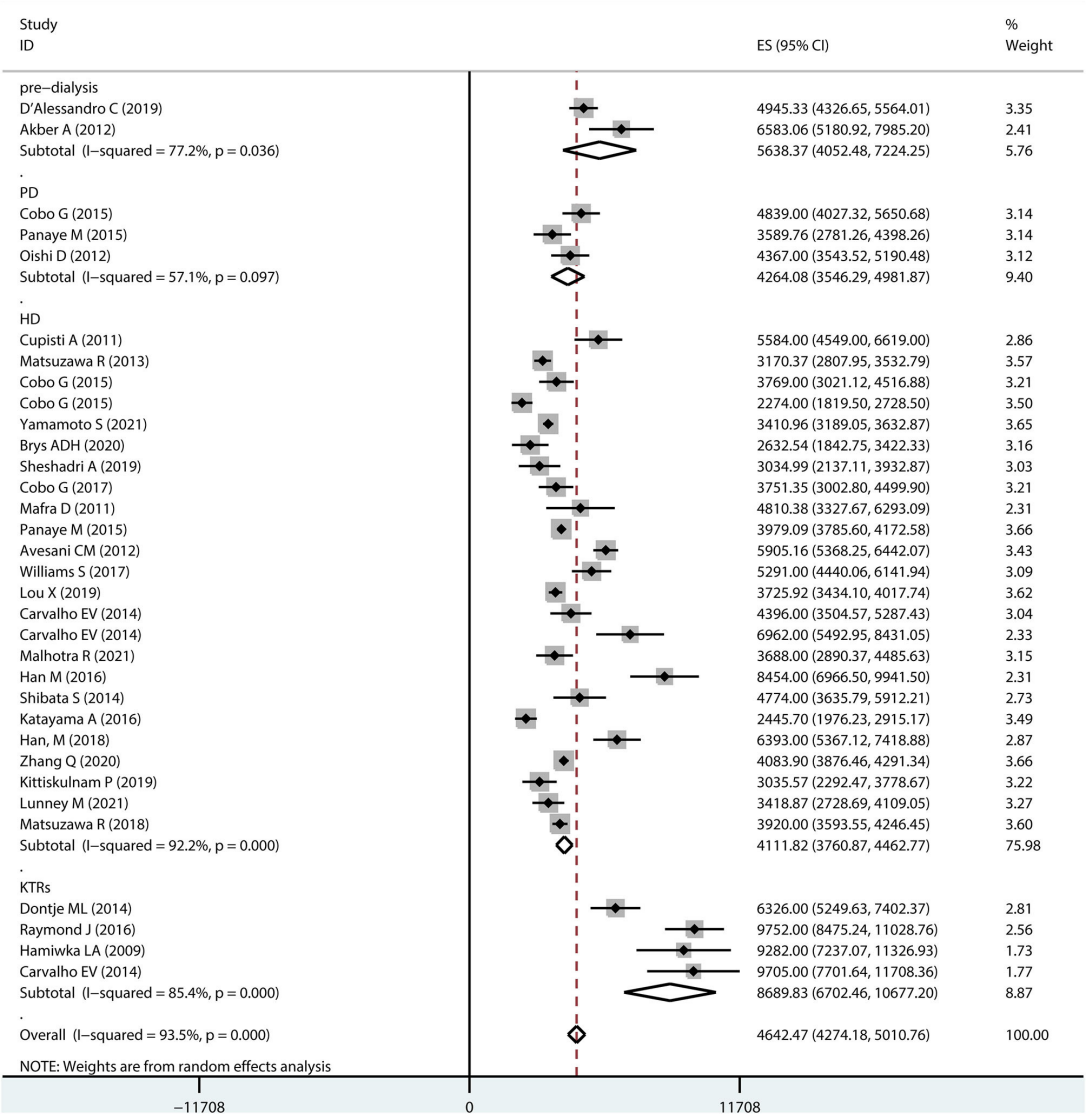
Forest plot of the overall step counts.

Figure 2 shows a forest plot of studies included in the meta-analysis. The results based on the random-effects model showed an overall step count of 4642.47 (95% *CI* 4274.18–5010.76) for patients with CKD. The considerable heterogeneity (*I*^2^ = 93.5%) may be a specific result of differences in patient age, stage of disease, and sample size.

Subgroup analysis was performed to determine the overall step counts in different disease stages. Two studies reported step counts for pre-dialysis (5638.37, 95% *CI* 4052.48–7224.25) patients. Three and 22 studies reported step counts for peritoneal dialysis (4264.08, 95% *CI* 3546.29–4981.87) and hemodialysis patients (4111.82, 95% *CI* 3760.87–4462.77), respectively. Furthermore, four studies reported step counts for kidney transplant recipients (8689.83, 95% *CI* 6702.46–10677.20).

### Daily Step Counts on Dialysis Days vs. Non-dialysis Days in Hemodialysis Patients

**Figure 3:**
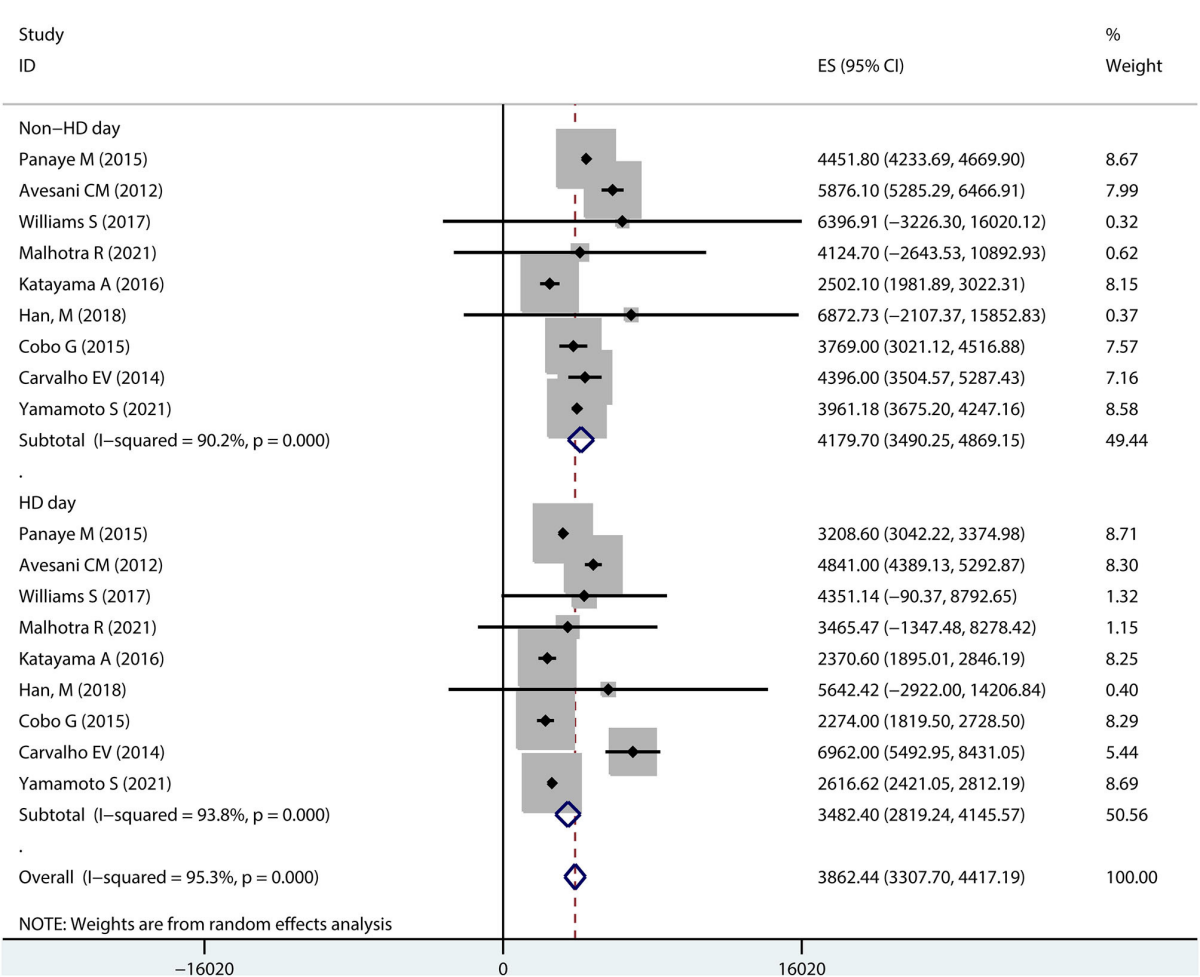
Forest plot of the overall step counts by dialysis and non-dialysis day.

Ten studies reported steps on dialysis days and non-dialysis days in hemodialysis patients (Figure 3). A pooled analysis showed that the overall step counts on a dialysis day was 3413.24 (95% *CI* 2825.61–4000.88), with high heterogeneity (*I*^2^ = 93.1%); while the overall step counts on a non-dialysis day was 4197.83 (95% *CI* 3631.92–4763.75), also with high heterogeneity (I-squared = 89.0%).

### Patients With CKD vs. Healthy Controls

**Figure 4:**
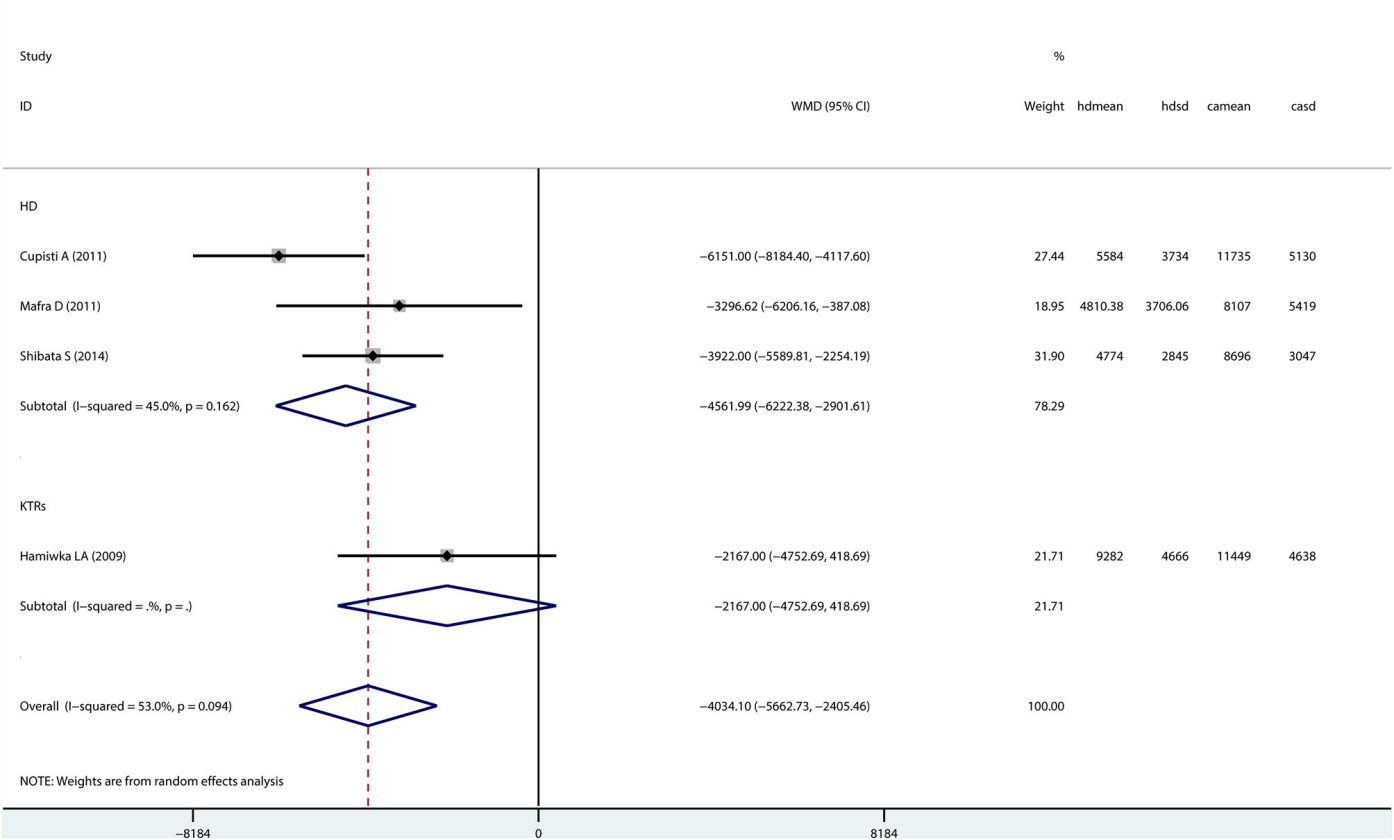
Forest plot of the overall step counts by CKD patients and healthy controls.

Only four studies (three hemodialysis patients and one kidney transplant recipient) evaluated step counts in patients with CKD compared with healthy controls (Figure 4). A random-effects model showed that step counts were lower in CKD patients than healthy controls (Mean difference: −4034.10; 95% *CI* −5662.73 to −2405.46), and the difference being statistically significant.

### Meta-Regression to Explore Heterogeneity for Step Counts in Patients With CKD

Table 2 shows the results of univariate and multivariate analyses. Univariate analyses showed that the daily step counts were higher across studies with those carried out in the Americas and among the kidney transplant recipients. Patients were aged <60 years had significantly higher step counts than patients aged ≥60 years. Therefore, these three variables (region, stage of CKD, and age) were eligible for inclusion in the multivariable regression analysis.

**Table 2:** Univariable and multivariable meta-regression analysis of step count in patients with CKD.

**Variable**	**No**.	**Univariable**			**Multivariable**		
		**Regression coefficient (95% *CI*)**	**SE**	***P*-value**	**Regression coefficient (95% *CI*)**	**SE**	***P*-value**
**Region**							
- Asian	8	1	–	–	1	–	–
- Europe	12	625.25 (−993.64, 2244.14)	792.69	0.436	232.83 (−963.88, 1429.54)	581.06	0.692
- Americas	13	2242.16 (625.75, 3858.56)	791.47	**0.008**	822.03 (−616.92, 2260.99)	698.68	0.250
**Stage of CKD**							
- Pre-dialysis	2	1	-	-	1	-	-
- PD	3	−812.86 (−2918.24, 1292.51)	1029.41	0.436	−659.74 (−2570.72, 1251.23)	927.87	0.484
- HD	24	−1689.75 (−4287.87, 908.36)	1270.33	0.194	−1681.84 (−3997.25, 633.58)	1124.24	0.147
- KTRs	4	3475.89 (887.1718, 6064.62)	887.17	**0.010**	2235.52 (−375.37, 4846.41)	1267.71	0.090
**Sample**							
- >100	9	1	–	–	NA	NA	NA
- ≤ 100	24	1170.28 (−341.74, 2682.29)	741.36	0.125	NA	NA	NA
**Bias**							
- score <7	8	1	–	–	NA	NA	NA
- score ≥ 7	25	−193.02 (−1868.68, 1482.63)	821.59	0.816	NA	NA	NA
**Age**							
- <60	14	1	–	–	1	–	–
- ≥60	18	−2056.08 (−3104.91, −1007.25)	513.56	**<0.001**	−1068.09 (−2310.34, 174.16)	603.17	0.089
**Measurement**							
- Accelerometer	6	1	–	–	NA	NA	NA
- Armband	7	60.33 (−2285.42, 2406.09)	1146.94	0.958	NA	NA	NA
- Pedometer	15	−424.77 (−2459.24, 1609.69)	994.74	0.673	NA	NA	NA
- Fitbit	5	446.38 (−2106.65, 2999.41)	1248.29	0.723	NA	NA	NA

*CKD, Chronic Kidney Disease; PD, Peritoneal Dialysis; HD, Hemodialysis; KTRs, Kidney Transplant Recipients; CI, Confidence Interval; SE, Standard Error; NA, Not Assess. Bold values indicate that the p-value < 0.05*.

The multivariable model was statistically significant [*R*^2^ = 51.12%; *F*_(6,25)_; *P* < 0.001] and reduced the *I*^2^ from 93.5 to 89.5%. However, there were no variables that remained statistically significant.

### Publication Bias

As shown in Supplementary Material 4, the contour-enhanced funnel plot was asymmetric. The Egger's test indicates a publication bias (*z* = 3.67, *P* = 0.004, Supplementary Material 5 provides the Egger's publication bias plot). Although it is usually impossible to know the precise mechanism for funnel plot asymmetry, publication bias could explain the presence of an asymmetrical funnel plot (26). No significant change in effect size was found when reanalyzing publication bias using the trim-and-fill computation (Supplementary Material 6).

## Discussion

**Figure 5:**
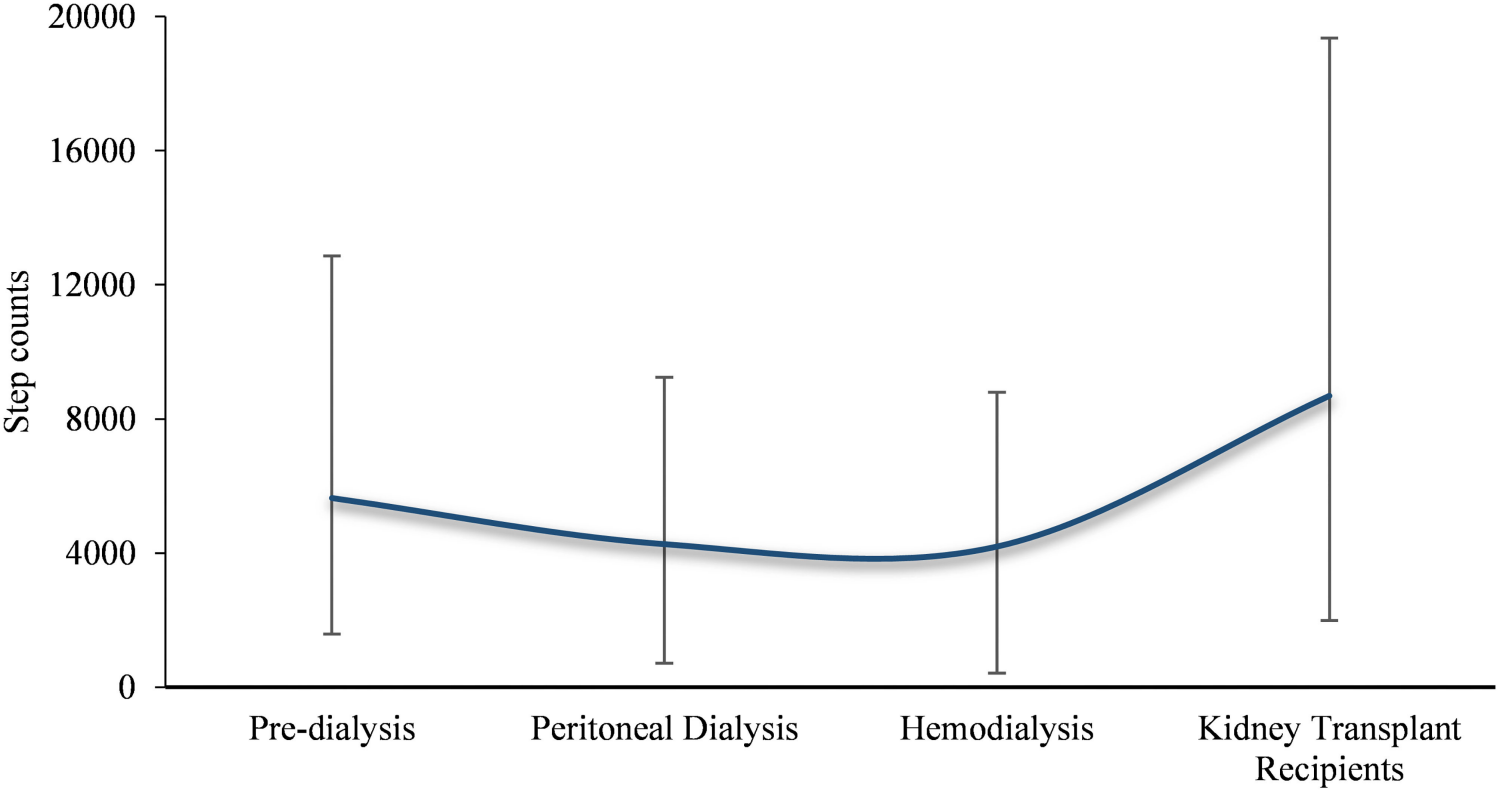
Trends in the number of steps taken by patients with CKD.

To our knowledge, this is the first systematic review of the literature to examine daily step counts in patients with the full spectrum of CKD. The key findings including (1) daily step counts in patients with CKD was 4642.47 (95% *CI*: 4274.18–5010.76); (2) patients with CKD have significantly lower daily step counts than healthy population, and the step counts began to decrease before dialysis, drops to a freezing point at the hemodialysis phase, and increases after kidney transplantation (Figure 5); (3) relatively high daily step counts in cases in the Americas or younger than 60 or kidney transplant recipients.

Physical activity is a cornerstone of renal rehabilitation in patients with CKD (42). Evidence suggests that regular physical activity is beneficial at all stages of CKD, including improvements in physical fitness, muscle functions, and health-related quality of life (43). More importantly, higher levels of physical activity are associated with a reduced risk of cardiovascular disease, all-cause mortality in this population (44). In comparing subjective methods (e.g., diaries, questionnaires) for physical activity assessment, activity monitors are regarded as the gold standard in detecting daily step counts and quantifying the volume of physical activity (45). A survey that included 5,656 participants reported that most CKD patients tend to lead sedentary and inactive lifestyles (46). A significantly higher risk of premature death has been reported in patients with <4,000 steps per day compared to hemodialysis patients with more than 4,000 steps per day (HR: 2.37; 95% *CI* 1.22–4.60) (40). A recent dose-response meta-analysis showed a 12% reduction in the risk of all-cause mortality for every 1,000 increase in step count (HR: 0.88; 95% *CI* 0.83–0.93) (47). However, most of the currently published studies were limited to assessing daily step counts taken by patients with CKD at a certain stage, and it would be helpful to pool different studies to understand trends to develop countermeasures to increase their physical activity.

In the current study, the average daily step counts for patients with CKD was 4642.47 (95% *CI* 4274.18–5010.76). The global physical activity recommendations issued by the American College of Sports Medicine (48) and the World Health Organization (49) recommend ~30 min of moderate physical activity per day as the minimum physical activity target value for maintaining and promoting health. Walking is recommended as the most basic physical activity for people with all types of chronic diseases. As a recommendation, Tudor-Locke et al. translated the above-recommended values (30 min or more of moderate physical activity per day) into a daily step count and recommended that patients with chronic diseases walk at least 7,000–10,000 steps per day (50). It can be seen that the physical activity level of CKD patients is much lower than recommended. Furthermore, a study by Chen et al. examined the relationship between walking and survival and renal replacement therapy in patients with CKD, reporting a 33% lower overall risk of death (HR: 0.67; 95% *CI*, 0.53–0.84) and a 21% lower risk of RRT (HR: 0.79; 95% *CI*, 0.73–0.85) (5). Additional studies have shown that walking is associated with an improved renal function (OR: 2.23; 95% *CI*, 1.91–2.60) in patients with CKD (51) and an inflammatory environment (52).

From the subgroup analysis of this study, the pooled analysis results further clarified the trend in the number of daily steps in patients with CKD at different stages, i.e., post-transplantation > pre-dialysis > peritoneal dialysis > hemodialysis. This result is similar to the findings of Wilkinson et al. on physical activity in patients with the whole spectrum of CKD (46). Predictably, physical activity levels increase with progressive physical function after kidney transplantation (53); for hemodialysis patients, the combination of dialysis-related fatigue, muscle atrophy, and other comorbidities (e.g., shortness of breath) further decreases physical activity over time in such patients (54). Only renal transplant recipients obtained daily step counts roughly equivalent to the recommended value, indirectly reflecting that most CKD patients do not reach the WHO recommended physical activity level. Therefore, further population-based research is needed to assess the impact of different measures on increasing walking to improve the prognosis of patients with CKD, which should be a priority in renal rehabilitation.

Moreover, elderly patients with CKD have a lower step count and should be prioritized. Frailty is a common comorbidity in CKD patients, and its prevalence increases with age (55). Data support the concept of a spiral of frailty, whereby elder patients with CKD are predisposed to inactivity and frailty (56), and frailty can cause further inactivity and disability (57). However, while disease and aging contribute to frailty, there are also potentially reversible factors, such as inflammation (58). Evidence that increased walking improves indicators of inflammation is well-established (59). Walking reflects physical activity in daily life in the elderly population (60); therefore, low step counts in elderly CKD patients suggest the urgency and need for further strengthening of walking management in this population.

We also noted that the assessment of step counts in CKD patients was mostly limited to the dialysis population, largely ignoring the pre-dialysis group, even though there were indications of physical inactivity already in the early stages of CKD. In this regard, of the 28 publications included, only four studies assessed step counts in pre-dialysis patients. Moreover, compared to healthy controls, step counts were at a disadvantage in dialysis patients and kidney transplant recipients. This result is expected, with a reduced step count associated with disease progression.

Based on a search of the literature, we obtained randomized control trials on increasing daily step counts in patients with CKD (Table 3). Overall, feasible strategies included: (1) pedometer-based intervention based on goal-setting theory; (2) physical activity increase based on exercise interventions to improve physical function; (3) changing the dialysis model. Different interventions can achieve their effect through different mechanisms of action.

**Table 3:** A summary of randomized control trials on increasing daily step counts in patients with CKD.

**References**	**Stage of CKD**	**Intervention**	**Findings**
Sheshadri et al. (61)	Hemodialysis	Providing pedometers in conjunction with weekly semi-scripted counseling sessions, patients were also set a goal of increasing their step count by 10% compared to the previous week.	After 3 months, patients in the intervention increased their average daily steps by 2,256 (95% *CI*, 978–3,537) more than the controls (*P* < 0.001).
Assawasaksakul et al. (62)	Hemodialysis	A 6-month intradialytic cycle ergometer for 60 min.	The physical activity in the exercise group was significantly increased from 5,613 to 8,725.1 steps/day in the sixth month (*P* = 0.046).
Martins et al. (63)	Hemodialysis	A 12-weeks moderate-intensity intradialytic resistance training	After 12 weeks, patients in the exercise group increased their average daily steps by 1,457.8 (95% *CI*, −232.6 to 3,148.2) than the controls (*P* = 0.22).
Pecoits et al. (64)	Hemodialysis	Patients receive high-volume online hemodiafiltration (HDF) or HD	Patients received HDF was +538 (95% *CI*, −330 to 1,407) steps/24 h compared with HD, but no statistically significant (*P* = 0.262).
O'Brien et al. (65)	KTRs	A multicomponent intervention combined SystemCHANGE™ + Activity Tracker	The intervention group increased daily steps at 3 months (mean difference, 608; standard error = 283, *P* = 0.03) compared to the control group.
Lyden et al. (66)	Predialysis	Patients received a Sit Less, Interact, Move More intervention.	Patients in the intervention group observed a significant increase in daily steps at week 20 (1265; 95 *CI*, 518–2012) but attenuated at week 24

There are some shortcomings of this study that should be mentioned. First, like other meta-analysis (67, 68), the statistical heterogeneity of the present meta-analysis was high and remained in the subgroup analysis, which is difficult to avoid in a meta-analysis of observational studies. For this result, we performed a meta-regression, heterogeneity may be associated with region, stage of disease, and age. However, a large portion of the high heterogeneity remains unexplained. Several other factors, such as duration of CKD disease, gender, etc., remain largely unknown and may be responsible for this unexplained heterogeneity. Secondly, although we conducted an exhaustive search of the three primary databases, the lack of non-English publications and unpublished studies may have contributed to the publication bias. We tried to account for this phenomenon by trim-and-fill in our analysis, but it remained unexplained. Thirdly, the current meta-analysis focused on literatures retrieved in standard databases, so gray literature and unpublished articles were not included. Although there may be additional studies of CKD patient step counts in the gray literature that may affect the estimation of pooled effects, we focused on the literature published with peer-reviewed outcomes.

## Conclusion

The current systematic review reveals the status of daily step counts in patients with CKD, which decreases with increasing CKD severity and increases after kidney transplantation. Although studies have begun to focus on strategies to improve step counts in patients with CKD, the inclusion of participants and the short follow-up period require further elucidation of the effects of long-term changes in physical activity in this population. In addition, future studies should focus more on step counts in pre-dialysis patients, and changing their physically inactive lifestyle early in the disease is central to slowing the progression of CKD.

## Data Availability Statement

The original contributions presented in the study are included in the article/Supplementary Material, further inquiries can be directed to the corresponding author.

## Author Contributions

FZ and YR: conceptualization and data analysis. YB and HW: retrieved literature and data extraction. FZ and LH: writing—original draft. LH: writing—review and editing. All authors have read and approved the final draft.

## Funding

This study was supported by the Third Batch of Specialist Nurse Education Program of Longhua Hospital (RC-2021-03-04).

## Conflict of Interest

The authors declare that the research was conducted in the absence of any commercial or financial relationships that could be construed as a potential conflict of interest.

## Publisher's Note

All claims expressed in this article are solely those of the authors and do not necessarily represent those of their affiliated organizations, or those of the publisher, the editors and the reviewers. Any product that may be evaluated in this article, or claim that may be made by its manufacturer, is not guaranteed or endorsed by the publisher.
