# Supplementary material for: Daily Step Counts in Patients With Chronic Kidney Disease: A Systematic Review and Meta-Analysis of Observational Studies
Source: Front Med (Lausanne). 2022 Feb 17;9:842423. doi: 10.3389/fmed.2022.842423 (PMC8891233; doi:10.3389/fmed.2022.842423)
Supplement: Supplementary file 1 [file Data_Sheet_1.zip › Supplementary File S1.docx]

**Supplementary File S1** Detail strategy for database

PubMed

("step per day" [Title/Abstract] OR "steps per day" [Title/Abstract] OR "step count" [Title/Abstract] OR “step/day" [Title/Abstract] OR "steps/day" [Title/Abstract] OR "step/d" [Title/Abstract] OR "steps/d" [Title/Abstract] OR "daily step" [Title/Abstract] OR "daily steps" [Title/Abstract]) AND ("Renal Insufficiency"[MeSH Terms] OR "Renal Replacement Therapy"[MeSH Terms] OR "Kidney Diseases"[MeSH Terms] OR "predialysis"[Title/Abstract] OR "pre-dialysis"[Title/Abstract] OR "CKF"[Title/Abstract] OR "CKD"[Title/Abstract] OR "CRF"[Title/Abstract] OR "CRD"[Title/Abstract] OR "kidney disease"[Title/Abstract] OR "renal disease"[Title/Abstract] OR "kidney failure"[Title/Abstract] OR "renal failure"[Title/Abstract] OR "renal insufficienc*"[Title/Abstract] OR "kidney insufficienc*"[Title/Abstract] OR "ESRF"[Title/Abstract] OR "ESKF"[Title/Abstract] OR "ESRD"[Title/Abstract] OR "ESKD"[Title/Abstract] OR "PD"[Title/Abstract] OR "HD"[Title/Abstract] OR "hemodialysis"[Title/Abstract] OR "haemodialysis"[Title/Abstract] OR "hemofiltration"[Title/Abstract] OR "haemofiltration"[Title/Abstract] OR "hemodiafiltration"[Title/Abstract] OR "haemodiafiltration"[Title/Abstract] OR "dialysis"[Title/Abstract] OR "Renal Transplantation"[Title/Abstract] OR "Kidney Grafting"[Title/Abstract] OR "Kidney Transplantation"[Title/Abstract] OR "KTRs"[Title/Abstract])

EMbase

("step per day":ti,ab,kw OR "steps per day":ti,ab,kw OR "step count":ti,ab,kw OR "step/day":ti,ab,kw OR "steps/day":ti,ab,kw OR "step/d":ti,ab,kw OR "steps/d":ti,ab,kw OR "daily step":ti,ab,kw OR "daily steps":ti,ab,kw) AND ('kidney failure'/exp OR 'renal replacement therapy'/exp OR 'kidney disease'/exp OR "predialysis":ti,ab,kw OR "pre-dialysis":ti,ab,kw OR "CKF":ti,ab,kw OR "CKD":ti,ab,kw OR "CRF":ti,ab,kw OR "CRD":ti,ab,kw OR "kidney disease":ti,ab,kw OR "renal disease":ti,ab,kw OR "kidney failure":ti,ab,kw OR "renal failure":ti,ab,kw OR "renal insufficienc*":ti,ab,kw OR "kidney insufficienc*":ti,ab,kw OR "ESRF":ti,ab,kw OR "ESKF":ti,ab,kw OR "ESRD":ti,ab,kw OR "ESKD":ti,ab,kw OR "PD":ti,ab,kw OR "HD":ti,ab,kw OR "hemodialysis":ti,ab,kw OR "haemodialysis":ti,ab,kw OR "hemofiltration":ti,ab,kw OR "haemofiltration":ti,ab,kw OR "hemodiafiltration":ti,ab,kw OR "haemodiafiltration":ti,ab,kw OR "dialysis":ti,ab,kw OR "Renal Transplantation":ti,ab,kw OR "Kidney Grafting":ti,ab,kw OR "Kidney Transplantation":ti,ab,kw OR "KTRs":ti,ab,kw)

Web of Science Core Collection

TS=("step per day" OR "steps per day" OR "step count" OR "step/day" OR "steps/day" OR "step/d" OR "steps/d" OR "daily step" OR "daily steps") AND TS=("predialysis" OR "pre-dialysis" OR "CKF" OR "CKD" OR "CRF" OR "CRD" OR "kidney disease" OR "renal disease" OR "kidney failure" OR "renal failure" OR "renal insufficienc*" OR "kidney insufficienc*" OR "ESRF" OR "ESKF" OR "ESRD" OR "ESKD" OR "PD" OR "HD" OR "hemodialysis" OR "haemodialysis" OR "hemofiltration" OR "haemofiltration" OR "hemodiafiltration" OR "haemodiafiltration" OR "dialysis" OR "Renal Transplantation" OR "Kidney Grafting" OR "Kidney Transplantation" OR "KTRs")
