# Supplementary material for: Daily Step Counts in Patients With Chronic Kidney Disease: A Systematic Review and Meta-Analysis of Observational Studies
Source: Front Med (Lausanne). 2022 Feb 17;9:842423. doi: 10.3389/fmed.2022.842423 (PMC8891233; doi:10.3389/fmed.2022.842423)
Supplement: Supplementary file 1 [file Data_Sheet_1.zip › Supplementary File S2.docx]

**Supplementary File S2** List of studies excluded at full text review stage and reasons for exclusion

| Author | Reasons |
| --- | --- |
| O'Brien et al. (2020) [1] | Trial |
| Zhou et al. (2020) [2] |  |
| Akber et al. (2014) [3] |  |
| Harris et al. (2021) [4] |  |
| Sullivan et al. (2019) [5] | Non-English |
| Tudor-Locke et al. (2009) [6] | Review |
| Rafik et al. (2019) [7] | Conference abstract |
| Alexander et al. (2001) [8] | Irrelevant to the research topic |

1. O'Brien T, Meyer T: **A Feasibility Study for Teaching Older Kidney Transplant Recipients How to Wear and Use an Activity Tracker to Promote Daily Physical Activity**. *Nephrol Nurs J* 2020, **47**(1):47-51.

2. Zhou H, Al-Ali F, Wang C, Hamad A, Ibrahim R, Talal T, Najafi B: **Harnessing digital health to objectively assess cognitive impairment in people undergoing hemodialysis process: The Impact of cognitive impairment on mobility performance measured by wearables**. *PLoS One* 2020, **15**(4):e0225358.

3. Akber A, Portale AA, Johansen KL: **Use of pedometers to increase physical activity among children and adolescents with chronic kidney disease**. *Pediatr Nephrol* 2014, **29**(8):1395-1402.

4. Harris AMY, Evans NM, Perez L, Burrows B, Derk GR, Chiu C, Wilund KR: **Working on Wellness: Impact of a Pilot Workplace Wellness Program in a Hemodialysis Center**. *Nephrol Nurs J* 2021, **48**(1):49-55.

5. Sullivan KM, Carter S, Jawa N, Harvey E, Amin R, Licht C: **Actigraphy assessment of sleep quality in children with end stage renal failure and dialysis**. *Nieren- und Hochdruckkrankheiten* 2019, **48**(3):108-109.

6. Tudor-Locke C, Washington TL, Hart TL: **Expected values for steps/day in special populations**. *Prev Med* 2009, **49**(1):3-11.

7. Rafik H, Aatif T, Bahadi A, Azizi M, Kabbaj DE: **Physical activity measured by pedometer in chronic hemodialysis patients**. *Science and Sports* 2019, **34**(6):381-387.

8. Alexander GC, Sehgal AR: **Why hemodialysis patients fail to complete the transplantation process**. *Am J Kidney Dis* 2001, **37**(2):321-328.
