# Supplementary material for: Daily Step Counts in Patients With Chronic Kidney Disease: A Systematic Review and Meta-Analysis of Observational Studies
Source: Front Med (Lausanne). 2022 Feb 17;9:842423. doi: 10.3389/fmed.2022.842423 (PMC8891233; doi:10.3389/fmed.2022.842423)
Supplement: Supplementary file 1 [file Data_Sheet_1.zip › Supplementary File S3.docx]

**Supplementary File S3** Risk of bias assessment of included studies

| **Studies** | **1. Were criteria for inclusion in the sample clearly defined?** | **2. Were the study subjects and the setting described in detail?** | **3. Was the exposure measured in a valid and reliable way?** | **4. Were objective, standard criteria used for measurement of the condition?** | **5. Were confounding factors identified?** | **6. Were strategies to deal with confounding factors stated?** | **7. Were the outcomes measured in a valid and reliable way?** | **8. Was appropriate statistical analysis used?** | **Score** |
| --- | --- | --- | --- | --- | --- | --- | --- | --- | --- |
| Cupisti A | Unclear | Unclear | Yes | Yes | Yes | Unclear | Yes | Yes | 5 |
| Matsuzawa R | Unclear | Yes | Yes | Yes | Unclear | Yes | Yes | Yes | 6 |
| Cobo G | Yes | Yes | Yes | Yes | Yes | Yes | Yes | Yes | 8 |
| Brys ADH | Yes | Yes | Yes | Yes | Yes | Yes | Yes | Yes | 8 |
| Sheshadri A | Yes | Yes | Yes | Yes | Yes | Yes | Yes | Yes | 8 |
| Cobo G | Yes | Yes | Yes | Yes | Yes | Yes | Yes | Yes | 8 |
| Yamamoto S | Yes | Yes | Yes | Yes | Yes | Yes | Yes | Yes | 8 |
| Mafra D | Unclear | Yes | Yes | Yes | Yes | No | Yes | Yes | 6 |
| Dontje ML | Yes | Yes | Yes | Yes | Yes | Yes | Yes | Yes | 8 |
| D'Alessandro C | No | Unclear | Yes | Yes | Unclear | Yes | Yes | Yes | 6 |
| Akber A | Yes | Yes | Yes | Yes | Yes | Yes | Yes | Yes | 8 |
| Panaye M | Yes | Yes | Yes | Yes | Unclear | Yes | Yes | Yes | 7 |
| Avesani CM | Unclear | Yes | Yes | Yes | Yes | Yes | Yes | Yes | 7 |
| Hamiwka LA | Yes | Unclear | Yes | Yes | Yes | No | Yes | Yes | 6 |
| Williams S | Yes | Yes | Yes | Yes | Yes | No | Yes | Yes | 7 |
| Lou X | Yes | Yes | Yes | Yes | Yes | Yes | Yes | Yes | 8 |
| Carvalho EV | Yes | Yes | Yes | Yes | Yes | No | Yes | Yes | 7 |
| Malhotra R | Yes | Unclear | Yes | Yes | Yes | No | Yes | Yes | 6 |
| Oishi D | Unclear | Unclear | Yes | Yes | Yes | No | Yes | Yes | 5 |
| Han M | Yes | Yes | Yes | Yes | Yes | No | Yes | Yes | 7 |
| Shibata S | Unclear | Unclear | Yes | Yes | No | No | Yes | Yes | 4 |
| Katayama A | Yes | Yes | Yes | Yes | Yes | No | Yes | Yes | 7 |
| Han M | Yes | Yes | Yes | Yes | Yes | No | Yes | Yes | 7 |
| Zhang Q | Yes | Yes | Yes | Yes | Yes | Yes | Yes | Yes | 8 |
| Kittiskulnam P | Yes | Yes | Yes | Yes | Yes | Unclear | Yes | Yes | 7 |
| Raymond J | Yes | Yes | Yes | Yes | Yes | Unclear | Yes | Yes | 7 |
| Lunney M | Yes | Yes | Yes | Yes | Yes | Yes | Yes | Yes | 8 |
| Matsuzawa R | Yes | Yes | Yes | Yes | Yes | Yes | Yes | Yes | 8 |
