# Supplementary figures and images for: Daily Step Counts in Patients With Chronic Kidney Disease: A Systematic Review and Meta-Analysis of Observational Studies
Source: Front Med (Lausanne). 2022 Feb 17;9:842423. doi: 10.3389/fmed.2022.842423 (PMC8891233; doi:10.3389/fmed.2022.842423)

**Supplementary File S4** The contour-enhanced funnel plots.


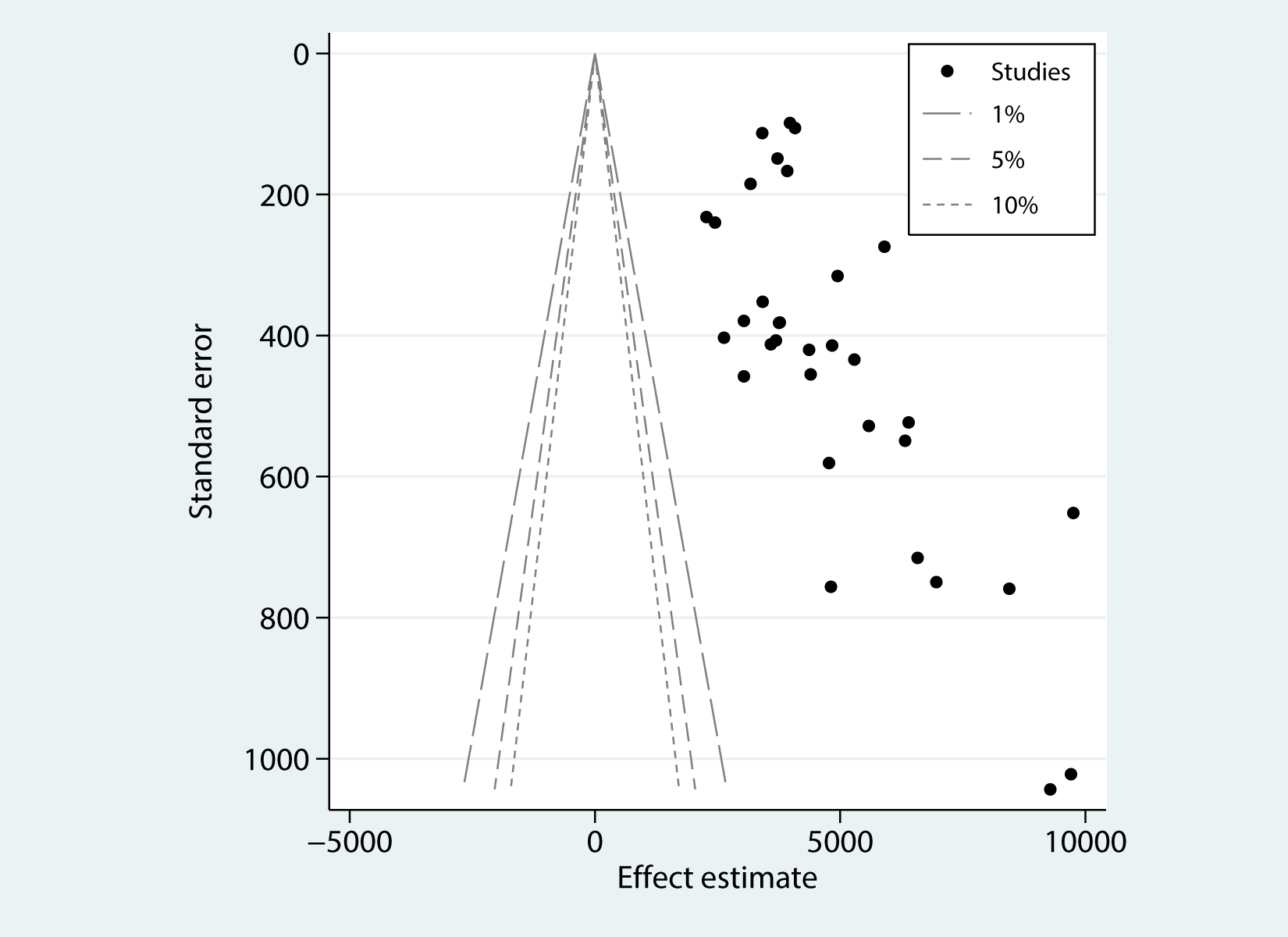

Supplement: Supplementary file 1 [file Data_Sheet_1.zip › Supplementary File S4.docx]

**Supplementary File S5** Egger’s publication bias plot


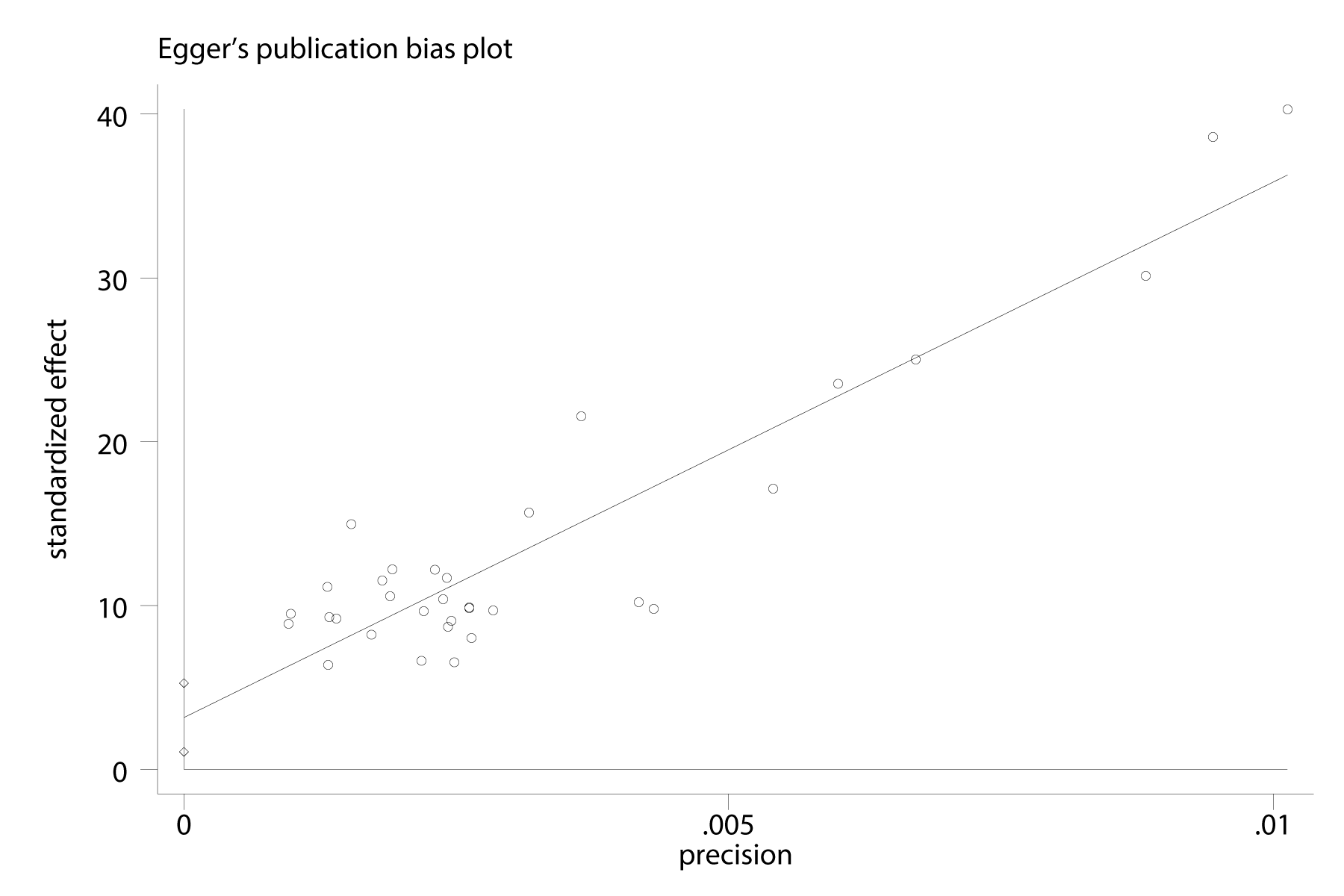


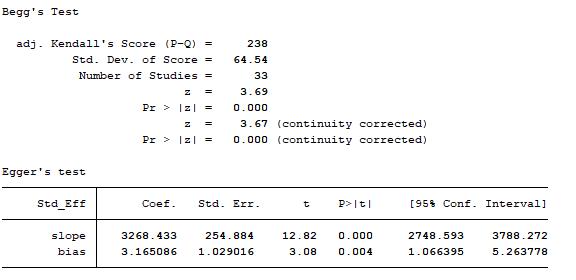

Supplement: Supplementary file 1 [file Data_Sheet_1.zip › Sypplementary File S5.docx]

**Supplementary File S6** Filled funnel plot with pseudo 95% CI


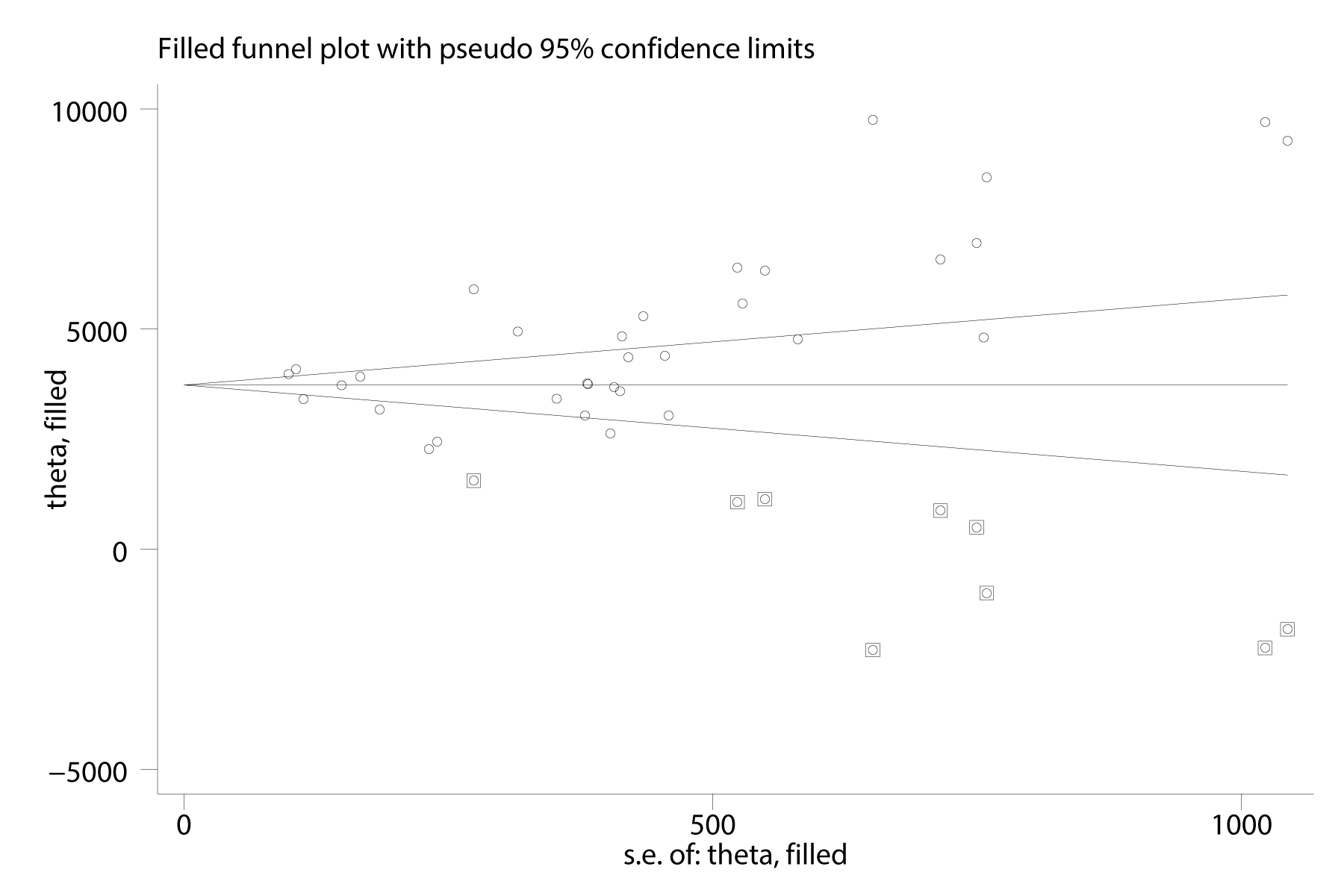


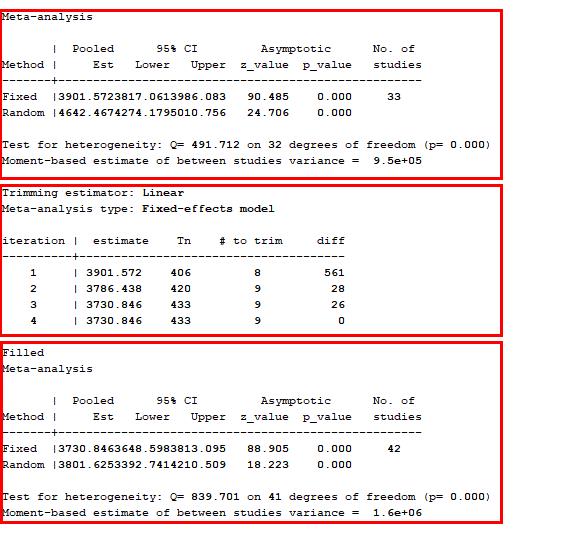

Supplement: Supplementary file 1 [file Data_Sheet_1.zip › Sypplementary File S6.docx]
